# Evaluation of Rv0220, Rv2958c, Rv2994 and Rv3347c of *Mycobacterium tuberculosis* for serodiagnosis of tuberculosis

**DOI:** 10.1111/1751-7915.12697

**Published:** 2017-02-20

**Authors:** Xiaolong You, Ranhui Li, Kanglin Wan, Liangzhuan Liu, Xiaoping Xie, Lanhua Zhao, Ning Wu, Xiangying Deng, Li Wang, Yanhua Zeng

**Affiliations:** ^1^ Institute of Pathogenic BiologyMedical CollegeUniversity of South ChinaHengyang421001China; ^2^ Hunan Provincial Key Laboratory for Special Pathogens Prevention and ControlHengyang421001China; ^3^ Hunan Province Cooperative Innovation Center for Molecular Target New Drug StudyHengyang421001China; ^4^ Clinical laboratoryThe First Affiliated Hospital of University of South ChinaHengyang421000China; ^5^ State Key Laboratory for Infectious Disease Prevention and Control/National Institute for communicable Disease Control and PreventionChinese Center for Disease Control and PreventionBeijing102206China; ^6^ Clinical laboratoryHengyang No.1 People's HospitalHengyang421001China

## Abstract

Tuberculosis (TB), the leading cause of death among infectious diseases worldwide, is caused by *Mycobacterium tuberculosis* (*M. tuberculosis*). Early accurate diagnosis means earlier prevention, treatment and control of TB. To confirm efficient diagnostic antigens for *M. tuberculosis*, the serodiagnosis value of four recombinant proteins including Rv0220, Rv2958c, Rv2994 and Rv3347c was evaluated in this study. The specificities and sensitivities of four recombinant proteins were determined based on enzyme‐linked immunosorbent assay (ELISA) by screening sera from smear‐positive pulmonary TB patients (*n* = 92), uninfected individuals (*n* = 60) and patients with *Mycoplasma pneumoniae* (*n* = 32) that potentially cross‐react with *M. tuberculosis*. The ELISAs showed that Rv0220, Rv2958c, Rv2994 and Rv3347c exhibited high specificities and sensitivities in detecting immunoglobulin G (IgG) antibody, with 98.3/91.3%, 91.7/85.9%, 93.3/89.1% and 93.3/80.4% respectively. According to the receiver‐operating characteristic (ROC) analysis, the area under the ROC of the target proteins was 0.988, 0.969, 0.929 and 0.945 respectively. Western blot was established to evaluate the immunoreactivities of target proteins to mice and human sera. Results demonstrated that Rv0220, Rv2958c, Rv2994 and Rv3347c could specifically recognize TB‐positive sera and the sera of mice immunized with the corresponding protein. Thus, Rv0220, Rv2958c, Rv2994 and Rv3347c were valuable potential diagnostic antigens for *M. tuberculosis*.

## Introduction

Tuberculosis (TB) is one of the leading chronic, progressive diseases caused by *Mycobacterium tuberculosis* (*M. tuberculosis*), which is threatening to human health and has popular epidemicity and high mortality rate (O'Garra *et al*., 2013). For the alarming increase in HIV/TB infection, the emergence of drug‐resistant strains and the increasing number of floating population, TB was thought to be one of the main factors that aggravated health burden on a global scale (Chetty *et al*., 2015). The World Health Organization (WHO) claimed that there were about average 8.5 million incident cases and 1.5 million deaths of TB all over the world per year during the past 3 years.

It is vital to make accurate early diagnosis for the prevention and control of TB. The lack of effective diagnostic tools is a major problem for accurate diagnosis of TB. The traditional methods for TB diagnosis were sputum smear and culture tests. *M. tuberculosis* culture was the gold standard method for diagnosis of TB. For the past few years, some new methods such as rapid culture, advanced imaging techniques, nucleic acid amplification (NAA) tests and T cell‐based interferon‐γ release have been improved (Ireton *et al*., 2010; Yun *et al*., 2016). However, these approaches were limited by the cost, scale, sensitivity and specificity (Bonnet *et al*., 2011). So far, there was no accurate and economic diagnostic tool to detect *M. tuberculosis*. The serological tests have been widely used to detect antibodies in clinical laboratories, which used recombinant proteins as antigen to screen TB (Pinto *et al*., 2012; Jiang *et al*., 2013). However, no ideal antigenic antigen can be used for serodiagnostic assays for TB, although these antigens could generate specific antibody in TB patients. Some certain *M. tuberculosis* proteins have been tested such as ESAT6, CFP10 (Hoff *et al*., 2016), CFP‐21 (Kalra *et al*., 2010), the 38 kDa antigen (Hur *et al*., 2015), Ag85 (Shin *et al*., 2008), Rv3872 (Mukherjee *et al*., 2007), Rv3873 (Liu *et al*., 2004) and Rv3117‐3121 (Zhang *et al*., 2012), which were mostly transmembrane proteins or secreted proteins of *M. tuberculosis*.

To exploit the ideal diagnostic antigens, we analysed all annotated genes in the *M. tuberculosis* genome using the web tools (http://genome.tbdb.org/) and the *M. tuberculosis* H37Rv database (tuberculist.epfl.ch). Those genes that are virulence‐related and essential for growth, metabolism and cell wall synthesis were selected, and then the B‐cell epitopes were predicted using seppa2.0 soft (http://lifecenter.sgst.cn/seppa/index.php) and analysed according to the Immune Epitope Database (IEDB). Based the above analysis, some antigens containing more B‐cell epitopes, including Rv0220, Rv2958c, Rv2994 and Rv3347c, were selected in this study. Besides, previous studies also showed that Rv0220 (cell surface esterase LipC), Rv2958c (PGL/p‐HBAD biosynthesis glycosyltransferase), Rv2994 (integral membrane protein) and Rv3347c (one of PPE family member, the C‐terminal sequence is Pro‐Pro‐Glu) exhibited immunogenicity (Singh *et al*., 2005; Gupta *et al*., 2010; Shen *et al*., 2012; Alvarez‐Corrales *et al*., 2013). These results implied that these antigens might be useful in the serodiagnosis of TB. Therefore, we evaluated the serodiagnosis value of Rv0220, Rv2958c, Rv2994 and Rv3347c in this study. The four genes coding corresponding proteins were amplified by polymerase chain reaction (PCR) respectively. Four recombinant expression plasmids including *pET‐28a‐Rv0220*,* pET‐28a‐Rv2958c*,* pET‐28a‐Rv2994* and *pET‐28a‐Rv3347c* were successfully constructed and transformed into *Escherichia coli* Rosetta (DE3). Four specific proteins were expressed as His‐tagged fusion proteins (46 kDa, 56 kDa, 38 kDa and 38 kDa) and purified by Ni‐NAT‐affinity chromatography. The sensitivities and specificities of four recombinant proteins were evaluated using sera from smear‐positive pulmonary TB patients (TB‐positive patients) and uninfected individuals (TB‐negative controls). As other respiratory infected pathogens such as *Mycoplasma pneumoniae* (*M. pneumoniae*) might have homogenous proteins with *M. tuberculosis*, sera obtained from *M. pneumoniae*‐infected patients were also evaluated for the analysis of sensitivities and specificities. Results in this study exhibited that all four target proteins were highly specific and sensitive for detecting *M. tuberculosis* infections. Thus, these proteins are promising antigens for a rapid and highly sensitive serodiagnostic test of TB.

## Results

### Target proteins were successfully expressed and purified

**Figure 1:**
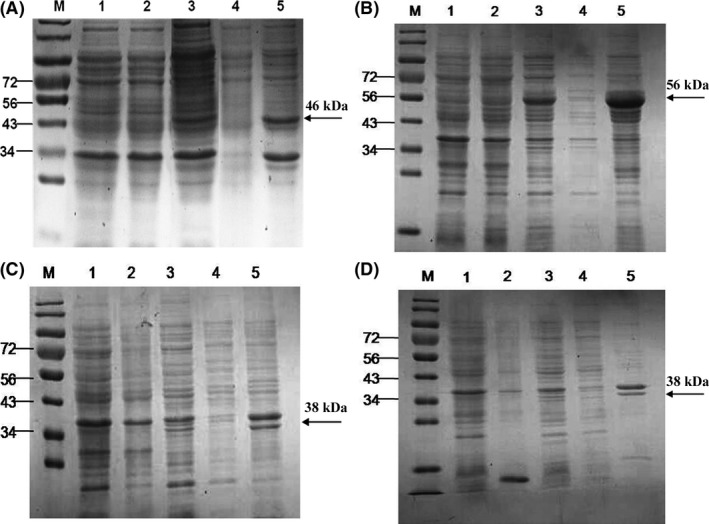
SDS‐PAGE analysis for the expressed recombinant proteins. Expressions of target proteins were analysed using SDS‐PAGE as described in materials and methods. A. Rv0220; B. Rv2958c; C. Rv2994; D. Rv3347c; M: protein marker (KDa). 1: induced bacteria with pET‐28a; 2: non‐induced bacteria with recombinant plasmid; 3: induced bacteria with recombinant plasmid; 4: supernatant of broken induced bacteria with recombinant plasmid; 5: precipitation of broken induced bacteria with recombinant plasmid.

**Figure 2:**
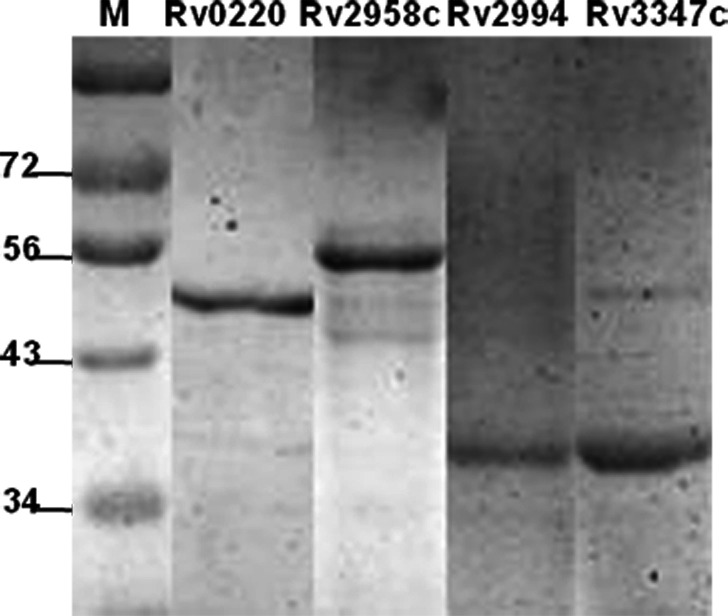
The purification of four recombinant proteins. Analyses of purified target proteins using SDS‐PAGE were performed as described in materials and methods. M: protein marker (KDa).

The target genes (*Rv0220*,* Rv2958c*,* Rv2994* and *Rv3347c*) were successfully amplified from genomic DNA of *M. tuberculosis* H37Rv strain. The recombinant prokaryotic vectors (*pET‐28a‐Rv0220*,* pET‐28a‐Rv2958c*,* pET‐28a‐Rv2994* and *pET‐28a‐Rv3347c*) were successfully constructed according to the results of PCR identification, enzyme‐cutting analysis and DNA sequencing (Figs S1 and S2). His‐tagged recombinant proteins were successfully expressed (Fig. 1) as inclusion bodies rather than soluble proteins, and then purified using Ni‐NTA beads (Fig. 2). These purified proteins that denatured in urea were renatured using renaturation solutions. After electrophoresis, grey scanning analysis showed that four target proteins accounted for more than 90% of the corresponding total proteins.

### Target proteins had good immunogenicities in mice

**Figure 3:**
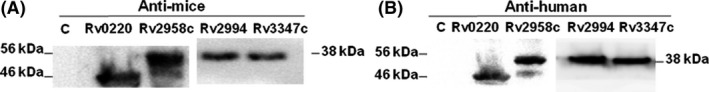
Western blot analysis of four proteins with different sera. Western blot analyses were performed as described in materials and methods. A. Western blot analysis of target proteins with mixed immunized mice sera. Proteins were used as coated antigen, antisera from immunized mice as primary antibody and anti‐mice IgG as secondary antibody. c: control sera from unimmunized mice. B. Western blot analysis of target proteins with mixed TB‐positive sera. Proteins were used as coated antigen, sera from clinical individuals as primary antibody and anti‐human IgG as secondary antibody. c: control sera from mixed TB‐negative individuals.

To evaluate the immunogenicity of target proteins (Rv0220, Rv2958c, Rv2994 and Rv3347c), sera collected from immunized mice were tested by Western blotting. As shown in Fig. 3A, strong specific reactions against corresponding recombinant proteins were observed for mice sera after three vaccinations, and no reaction was observed for unimmunized mice sera from the control group. The sera IgG antibodies of immunized mice were, respectively, detected by corresponding protein‐based ELISAs. The antibody titres of immunized mice sera were 1:6400, 1:12 800, 1:12 800 and 1:6400 respectively (data not shown). These results indicated that target proteins Rv0220, Rv2958c, Rv2994 and Rv3347c had good immunogenicities and could stimulate mice to produce high titres of specific antibodies.

### Target proteins exhibited excellent immunoreactivities to human sera

To further verify whether four target proteins could react with different clinical sera, Western blot assays were performed using mixed TB‐positive sera and TB‐negative sera. Results showed that Rv0220, Rv2958c, Rv2994 and Rv3347c could recognize specially the mixed TB‐positive sera but not TB‐negative sera (Fig. 3B), which demonstrated that Rv0220, Rv2958c, Rv2994 and Rv3347c exhibited excellent immunoreactivities.

### Target proteins had high sensitivities and specificities in serodiagnosis of TB

**Figure 4:**
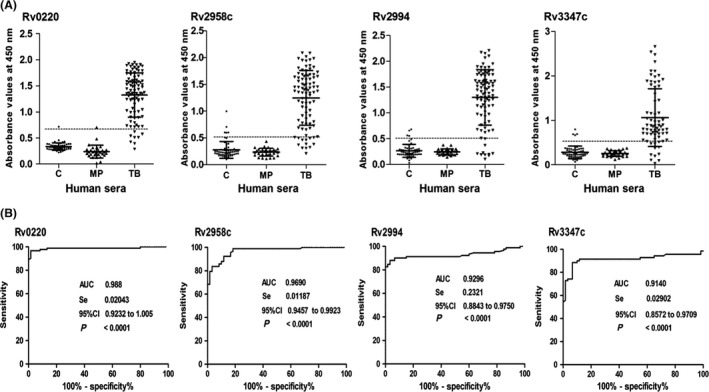
Reactivities of sera from clinical individuals to target proteins and the ROC curve. ELISA reactivities of target proteins with different clinical sera and the receiver‐operating characteristic (ROC) curve were evaluated with the panel of different sera samples. A. ELISA analysis of target proteins with different clinical sera. ELISA experiments were performed as described in Methods. B. The ROC curves of four target proteins. The corresponding ROC curves for four target proteins were produced using graphpad prism 5 according to the distribution of the optical density in ELISA test. And the area under the curve (AUC), standard error (SE), sensitivity and specificity with 95% confidence intervals (95% CI) and *P* value were indicated. C. TB‐negative sera control; TB, TB‐positive sera; MP, MP‐positive sera; Mp, *M. pneumoniae*.

The sensitivities and specificities of the target proteins for the diagnosis of TB were evaluated by indirect ELISA. Results demonstrated that four recombinant proteins could react with most of sera from TB‐positive patients (*n* = 92), whereas only few TB‐negative sera and *M. Pneumoniae‐*positive sera could react with recombinant proteins, which demonstrated high sensitivities and specificities of target proteins. The cut‐off values from the sera of 60 TB‐negative individuals were determined as 0.701 for Rv0220, 0.554 for Rv2958c, 0.548 for Rv2994 and 0.591 for Rv3347c respectively. The sensitivities were 91.3% for Rv0220, 85.9% for Rv2958c, 89.1% for Rv2994 and 80.4% for Rv3347c respectively (Fig. 4A, Table 1). And the specificities were 98.3% for Rv0220, 91.7% for Rv2958c, 93.3% for Rv2994 and Rv3347c for 93.3%. According to the ROC analysis, the AUC were 0.988 for Rv0220, 0.969 for Rv2958c, 0.929 for Rv2994 and 0.945 for Rv3347c, respectively (Fig. 4B), which demonstrated that the ELISA results were credible and Rv0220, Rv2958c, Rv2994 and Rv3347c had high sensitivities and specificities in serodiagnosis of TB.

**Table 1:** Sensitivities and specificities of ELISA in the detection of TB

Protein	Sensitivity TB‐positive patients	Specificity TB‐negative individuals	Specificity Mp‐positive patients
Rv0220^a^	91.3% (84/92)	98.3% (59/50)	96.80% (31/32)
Rv2958c^a^	85.9% (79/92)	91.7% (55/60)	100.0% (32/32)
Rv2994^a^	89.1% (82/92)	93.3% (56/60)	100.0% (32/32)
Rv3347c^a^	80.4% (74/92)	93.3% (56/60)	100.0% (32/32)

a The cut‐off values were calculated to be the mean OD value plus two standard deviations (SDs) for TB‐negative individuals.

## Discussion

Tuberculosis is one of the lethal infectious diseases caused by *M. tuberculosis*. The best prognosis for TB rests with the early diagnosis for *M. tuberculosis* infection. However, the existing diagnosis methods were limited by some disadvantages such as high cost, low sensitivity and specificity. Compared with traditional bacterial culturing, ELISA is a commonly used serological method due to its high sensitivity and specificity, low cost and other advantages. The identification of valuable *M. tuberculosis* antigens is vital to the improvement of serodiagnostic methods of TB. In recent years, comparative genomic studies have identified some promising recombinant proteins for TB serodiagnosis such as ESAT‐6, CFP‐10, Hsp60 (Shekhawat *et al*., 2016), PE35 (Baumann *et al*., 2014), PPE57 (Chen *et al*., 2009), PPE68 (Pourakbari *et al*., 2015), Rv1168c, Rv1985c and Rv3878 (Dosanjh *et al*., 2016). The combination of antigens ESAT‐6 and CFP‐10 has been used to identify individuals with active TB in commercial tests. The specificities of some tests might be higher than 90%, but the sensitivities were unsatisfactory. Therefore, there was still no suitable test to meet the needs of high specificity and strong sensitivity at the same time, and it was also hard to distinguish between TB‐positive individuals and BCG‐vaccinated individuals. There is an urgent need to find more efficient diagnostic antigens that can overcome the disadvantages as described above.

According to the bioinformatics analysis for all annotated genes in *M. tuberculosis* genome using the tbdb tools and *M. tuberculosis* H37Rv database combination with the B‐cell epitope prediction using seppa2.0 soft and the Immune Epitope Database (IEDB), some antigens containing more B‐cell epitopes, including Rv0220, Rv2958c, Rv2994 and Rv3347c, were selected in this study to evaluate the potential serodiagnostic value. And previous studies have verified that Rv0220 was a cell‐surface‐associated esterase of *M. tuberculosis*, which was highly immunogenic and could elicit production of both antibodies and cytokines/chemokines. Rv2958c and Rv3347c were recognized and defined by IFN‐γ production in blood from TB‐negative individuals exposed to *M. tuberculos*is; IFN‐γ‐mediated responses showed strong cellular recognition of Rv2958c and Rv3347. Rv2994 encoded by efflux pump genes has been demonstrated in multidrug‐resistant isolates (Singh *et al*., 2005; Gupta *et al*., 2010; Shen *et al*., 2012; Alvarez‐Corrales *et al*., 2013). There is no report about the serological diagnosis potential of four proteins so far.

In this study, the genes *Rv0220*,* Rv2958c*,* Rv2994* and *Rv3347c* were successfully cloned and the corresponding recombinant proteins Rv0220, Rv2958c, Rv2994 and Rv3347c were expressed and then purified by Ni‐NAT affinity chromatography. The renaturation of denatured proteins was performed in a renaturation buffer. Furthermore, ELISA results suggested that each target protein could react with sera from corresponding immunized mice. In addition, sera from the corresponding immunized mice could specifically recognize each recombinant protein by the results of Western blot assay. These results demonstrated that the recombinant proteins have excellent specific immunoreactivity with sera from immunized mice.

Detecting the antibodies of *M. tuberculosis* using target proteins as antigens showed obvious advantages such as high specificity, high sensitivity, simple and time‐saving. In our study, ELISA results showed that most of sera from TB‐positive patients (*n* = 92) had good reactivities with four recombinant proteins, which demonstrated high sensitivities of target proteins. In addition, only few sera from 60 TB‐negative individuals and 32 individuals with *M. pneumoniae* infection could react with recombinant proteins, which demonstrated high specificities of target proteins. Besides, the Western blot analysis showed that mixed TB‐positive sera yielded positive reactivities to all recombinant proteins, but TB‐negative sera could not react with recombinant proteins, which indicated that these recombinant proteins could be used as diagnostic antigens for screening TB patients. Further studies need to be performed before Rv0220, Rv2958c, Rv2994 and Rv3347c were commercially used for the serodiagnosis of TB in clinical laboratory. Therefore, we will expand the number of clinical samples to better evaluate the serodiagnostic value of these antigens for the diagnosis of TB.

In summary, we assessed four recombinant candidates including Rv0220, Rv2958c, Rv2994 and Rv3347c, which exhibited high sensitivity (91.3%, 85.9%, 89.1% and 80.4%) and high specificity (98.3%, 91.7%, 93.3% and 93.3%) for the detection of *M. tuberculosis* infection. These results highlighted that the recombinant proteins Rv0220, Rv2958c, Rv2994 and Rv3347c are promising antigenic markers of TB. More sera samples need to be performed before these recombinant proteins can be definitively verified available in the serodiagnosis of TB.

## Materials and methods

### Human sera

This study was approved by the Human Ethics Committee of the University of South China. All participants have signed informed consent form. A total of 92 TB‐positive sera samples (*n* = 92) were randomly selected (age range, 1–85 years), including 67 sera samples from smear‐positive for acid‐fast bacilli and culture‐positive pulmonary TB patients and 25 sera samples from smear‐negative but culture‐positive pulmonary TB patients. TB‐negative control subjects (*n* = 60; age range, 20–63 years) from individuals who had negative chest X‐rays and sputum culture negative for *M. tuberculosis*. All sera samples were obtained from the third people's hospital of Hengyang City, the specialized TB hospital, Hunan, China. *M. pneumonia*‐positive sera samples (*n* = 32; age range, 19–81 years) from *M. pneumonia‐*infected individuals were verified by clinical manifestations and the serological detection, which were collected from the First Affiliated Hospital, University of South China.

### Cloning, expression and purification of target proteins

The ORFs corresponding to *Rv0220*,* Rv2958c*,* Rv2994* and *Rv3347c* (the gene segment 6238‐7278 bp was selected) were amplified by PCR from *M. tuberculosis* H37Rv genomic DNA respectively. The genomic DNA of *M. tuberculosis* H37Rv strain was a gift from Chinese Center For Disease Control And Prevention (CDC, Beijing, China). *Escherichia coli* Rosetta (DE3) and the prokaryotic expression vector *pET28a* (Novagen, Madison, WI, USA) were previously conserved in our laboratory. The corresponding primers, restriction endonucleases, annealing temperature and amplicon size of four target genes for amplification are shown in Table 2. PCR products were, respectively, digested with corresponding restriction endonucleases and then cloned into expression vector *pET*‐*28a*, which contains His‐tag in the C‐terminal. The recombinant vectors confirmed by PCR, restriction enzyme identification and sequence analysis were transformed into the *E. coli* Rosetta prokaryotic expression system and then cultured in Luria–Bertani medium containing 50 μg ml^−1^ of kanamycin. After the bacterial absorbance value at 600 nm reached 0.6‐0.8, Rv0220 was expressed at 37°C for 3 h with 2.0 mM isopropyl‐B‐D‐thiogalactopyranoside (IPTG). Similarly, Rv2958c was expressed at 37°C for 6 h with 1.5 mM IPTG, Rv2994 at 28°C for 12 h with 1.5 mM IPTG and Rv3347c at 37°C for 8 h with 1.5 mM IPTG. These His‐tagged proteins were purified by affinity chromatography on a Ni‐nitrilotriacetic acid (NTA) agarose column (Qiagen, Chatsworth, CA, USA) using fast protein liquid chromatography system (GE, AKTA, Massachusetts, USA), Briefly, precipitation of broken induced bacteria with recombinant plasmid were obtained, washed, resuspended and then added into NTA agarose column. After being washed with 50 mM Tris–HCl, 500 mM NaCl, 50 mM imidazole, 6 M urea (pH = 8.0) and eluted with 50 mM Tris–HCl, 500 mM NaCl, 500 mM imidazole and 6 M urea (pH = 8.0), these proteins were then renatured using renaturation solutions (50 mM NaCl, 0.5 mM EDTA, 50 mM Tris, 0.2 mM DTT, 1 mM GSH, 0.1 mM GSSG, 35% glycerol, pH8.35). After ultrafiltration and concentration, the concentrations of target proteins were determined using a bicinchoninic acid (BCA) protein assay kit (Pierce, Rockford, IL, USA).

**Table 2:** Primers and enzymes used for cloning of four target proteins

Gene	Forward primer^a^ (5′–3′) (above) Reverse primer^a^ (5′–3′) (blow)	Restriction endonuclease	Annealing temperature (°C)	Amplicon size (bp)
*Rv0220*	CCGAATTCATGAACCAGCGACGCGC CCGCTCGAGTTAGATGACCTCTITCGCG	*EcoRI* *Xhol*	55	1212
*Rv2958c*	AAGAATTCATGGAGGAAACAAGCGTCGC AACTCGAGTTAGCAGACGAGCCGCAG	*EcoRI* *Xhol*	46	1287
*Rv2994*	CGCGGATCCATGTCGCGAGATCCGACTG CCCAAGCTTTCATGTCGGTGGCGTTATTG	*BamHI* *Hind III*	59	1338
*Rv3347c*	CGGGATCCGACATAGACGGCCAGATTGAC CCCAAGCTTGTTGCCTCGCCACAAGATG	*BamHI* *Hind III*	61	1044

a Each restriction site used.

### Immunization of mice with the recombinant proteins

Eight specific pathogen‐free (SPF)‐grade female BALB/c mice (age 6–8 weeks, weight 19–24 g) were immunized by subcutaneous injection with 30 μg of each purified recombinant protein, which emulsified in incomplete Freund's adjuvant (complete Freund's adjuvant for the first time) (Sigma, St. Louis, MO). The mice were immunized three times at biweekly intervals. The sera prior to the initial immunization were used as negative controls prior to the initial immunization, and antisera (100 μl) against recombinant proteins were collected the day before each immunization. Ethical approval was given by Institutional Animal Care and Use Committee, University of South China, and all procedures were carried out according to the guidelines.

### ELISAs

Each purified recombinant protein was diluted in 0.1 M carbonate buffer (0.1 M Na_2_CO_3_, 0.1 M NaHCO_3_; pH = 9.6) to a concentration of 10 μg ml^−1^. 96‐well microtiter plates (Costar, Corning, NY, USA) were filled with 100 μl of the above diluted recombinant proteins per well at 37°C for 2 h. After washing three times with PBS, plates were then blocked with 5% (w/v) skim milk diluted in PBS for 2 h at 37°C. After being washed as described above, antisera from immunized mice (1:200) or sera from clinical individuals (1:100) were diluted in blocking buffer and added to each well and then incubated for 1 h at 37°C. After being washed five times with PBST, 50 μl of HRP‐conjugated anti‐mice IgG (1:10 000) or HRP‐conjugated anti‐human IgG (1:20 000) was added to each well and incubated at 37°C for 1 h followed by washing three times; 50 μl per well of tetramethylbenzidine (TMB) liquid was added to each well and incubated for 15 min in a dark place. The colour reactions were terminated by adding 100 μl of 2 M sulfuric acid. The absorbance values were determined at 450 nm using an ELISA microplate reader (Bio‐Rad). All samples were performed in duplicate.

### Western blot analysis

Each recombinant protein (45 μl) was diluted by sample loading buffer (15 μl) and boiled at 100°C for 10 min. All recombinant proteins were then separated on 12% sodium dodecyl sulfate–polyacrylamide gel electrophoresis (SDS‐PAGE) gels and transferred to polyvinylidene fluoride (PVDF) membranes using semidry Trans‐Blot SD (Bio‐Rad, Corning Inc., NY, USA) system. PVDF Membranes were blocked at 37°C water‐jacket incubator for 2 h with 5% (w/v) skim milk diluted in phosphate‐buffered saline (PBS) and then washed 3 times with PBST (PBS containing 0.05% Tween‐20). After being washed three times, PVDF membranes were incubated with mice sera (1:200) or mixed TB‐positive sera (1:100) diluted in PBST. After being washed, PVDF membranes were incubated at 37°C for 1 h with horseradish peroxidase (HRP)‐conjugated anti‐mouse IgG (1:20 000) (ab6728, Abcam) or HRP‐conjugated anti‐human IgG (1: 20 000) (ab6858, Abcam) respectively.

### Statistical analysis

graphpad prism 5 software was used for analysis of the data. The cut‐off values were presented as the geometric mean OD for the TB‐negative individual sera samples plus 2 SD. Specificity and sensitivity were calculated according to the following formulas: specificity = number of true negatives/(number of true negatives + number of false positives) and sensitivity = number of true positives/(number of true positive + number of false negatives). Difference was statistically significant at *P* values < 0.05. And the receiver‐operating characteristic (ROC) curves were created to reflect the relationship between sensitivity and specificity. When the area under the ROC curve (AUC) is between 0 and 1, it indicates that the ELISA results are credible.

## Conflict of interest

None declared.

## Supporting information


**Fig. S1.** Electrophoretic analysis of PCR products of four genes.
**Fig. S2.** Electrophoretic analysis of *pET‐28a*/*Rv0220*,* pET‐28a*/*Rv2958c*,* pET‐28a*/*Rv2994* and *pET‐28a*/*Rv3347c* by PCR (A) and restriction enzyme digestion (B).
