# Corrigendum

**DOI:** 10.1111/1751-7915.12743

**Published:** 2017-06-22

You, X., Li, R., Wan, K., Liu, L., Xie, X., Zhao, L., *et al*. (2017) Evaluation of Rv0220, Rv2958c, Rv2994 and Rv3347c of *Mycobacterium tuberculosis* for serodiagnosis of tuberculosis. *Microb Biotechnol* 10: 604–611.

In the original publication (Microbial Biotechnology, 2017; 10: 604–611), the affiliations for two of the authors were incorrect. The affiliations should read as follows:

“Xiaopeng Xie, *Clinical laboratory, The First Affiliated Hospital of University of South China, Hengyang, 421000, China*”

And

“Ning Wu, *Clinical laboratory, Hengyang No.1 People's Hospital, Hengyang, 421001, China*”

The authors sincerely apologize for the confusion caused by this error.

