# Supplementary material for: Evaluation of Rv0220, Rv2958c, Rv2994 and Rv3347c of Mycobacterium tuberculosis for serodiagnosis of tuberculosis
Source: Microb Biotechnol. 2017 Feb 20;10(3):604–11. doi: 10.1111/1751-7915.12697 (PMC5404193; doi:10.1111/1751-7915.12697)
Supplement: Supplementary file 1 — Fig. S1. Electrophoretic analysis of PCR products of four genes. Fig. S2. Electrophoretic analysis of pET‐28a/Rv0220, pET‐28a/Rv2958c, pET‐28a/Rv2994 and pET‐28a/Rv3347c by PCR (A) and restriction enzyme digestion (B). [file MBT2-10-604-s001.docx]

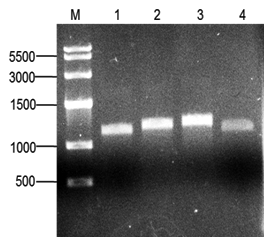


Fig. 1 Electrophoretic analysis of PCR products of four genes.

M: DNA Marker; 1: *Rv0220* PCR product; 2: *Rv2958c* PCR product;

3: *Rv2994* PCR product; 4: *Rv3347c* PCR product.


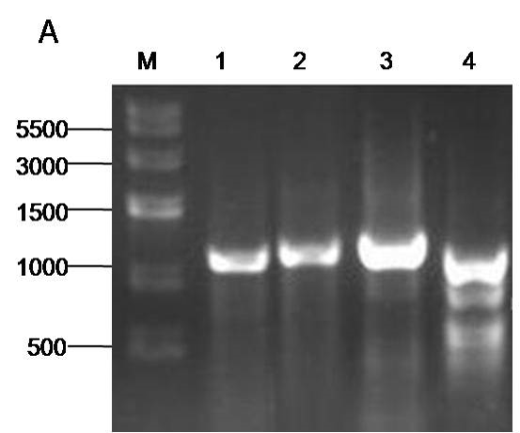

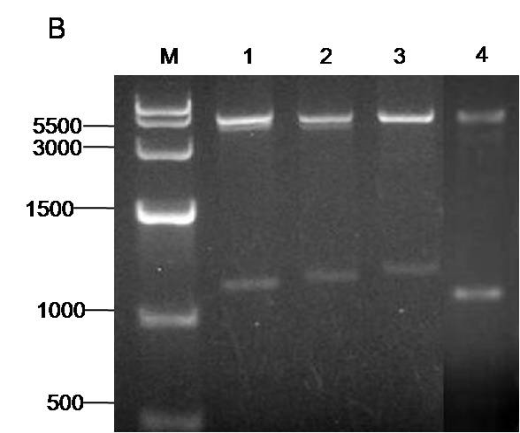


Fig. 2 Electrophoretic analysis of *pET-28a*/*Rv0220*, *pET-28a*/*Rv2958c*, *pET-28a*/*Rv2994* and *pET-28a*/*Rv3347c* by PCR (A) and restriction enzyme digestion (B).

M: DNA Marker; 1: *pET-28a*/*Rv0220*; 2: *pET-28a*/*Rv2958c*;

3: *pET-28a*/*Rv2994*; 4: *pET-28a*/*Rv3347c*
